# Embedding routine hearing health checks within existing Meals on Wheels services – A protocol for the SOUND-BITES Program pilot study

**DOI:** 10.1371/journal.pone.0354082

**Published:** 2026-07-14

**Authors:** Diana Tang, Jessica Turner, Ugochukwu Eze, John Newall, Kompal Sinha, Carolyn Mee, Claudia Odello, Andrew Blaikie, Bamini Gopinath

**Affiliations:** 1 Macquarie University Hearing Research Centre, Macquarie University, Sydney, Australia; 2 School of Health Sciences and Nursing, Macquarie University, Sydney, Australia; 3 School of Medicine, University of St Andrews, St Andrews, United Kingdom; 4 Department of Linguistics, Macquarie University, Sydney, Australia; 5 Department of Economics, Macquarie University, Sydney, Australia; 6 Sound Scouts, Sydney, Australia; 7 Meals on Wheels NSW, Sydney, Australia; Manipal Academy of Higher Education, INDIA

## Abstract

**Introduction:**

There is a high prevalence of undiagnosed and untreated hearing loss among older adults. Due to finite resources, task shifting to trained non-specialists is a strategy to improve equity of access to hearing health care. This pilot study aims to implement and evaluate a novel model of hearing care known as the SOUND-BITES program. This will leverage evidence-based mobile health technologies (Arclight otoscopy and Sound Scouts hearing screening app) and partnerships with Meals on Wheels New South Wales and Master of Clinical Audiology students.

**Methods:**

SOUND-BITES provides a hearing health assessment involving otoscopy using Arclight, hearing screening using the Sound Scouts tablet-based app, and hearing health education to consenting Meals on Wheels clients and their household members. Volunteers from Meals on Wheels and audiology students will be trained to deliver the screening, education and referral components of this program. This pilot study utilises a mixed-methods approach to evaluation. The primary outcomes are program acceptability and feasibility; and secondary outcomes are actions taken by clients to address hearing loss, changes in hearing handicap and cost-benefits six months after program completion. Quantitative data will be collected from a pre- and post-program survey, and qualitative data from an optional post-program interview with clients and volunteers. Findings from the project will be disseminated through peer-reviewed journals and conferences as well as to relevant communities including the Meals on Wheels network.

## Introduction

One in five people have hearing loss globally and this prevalence is expected to rise to one in four by 2050 due to ageing demographics [1]. Despite the availability of effective diagnostic tools and hearing device interventions, adults delay seeking help for their hearing loss by an average of 8.9 years [2]. This delay is caused by numerous barriers including hearing loss-related stigma; hearing screening not being systematically implemented in adults; and lack of public awareness and prioritisation of hearing loss [3]. When left untreated, hearing loss is associated with an increased risk of loneliness, depression, frailty, and falls [4–10]. Hearing loss in midlife (18–64 years) is also suggested to be the single largest modifiable risk factor for a future dementia diagnosis [11]. Early detection of hearing loss and appropriate interventions can prevent these associated adverse effects in older people [3,12]. These interventions can range from hearing devices such as hearing aids and cochlear implants to practical environmental modifications like reducing background noise and using tablecloths and carpet to minimise reverberations [13]. However, the benefits of these interventions cannot be realised if hearing loss goes undetected, and constraints around the availability of and access to hearing healthcare specialists goes unaddressed.

The use of trained nonspecialist providers to close the gap in healthcare access and improve outcomes in vulnerable communities, is a growing approach in both low to middle- and high-income countries [14,15]. This ‘task shifting’ from specialist providers to non-specialist delivered care has been identified by the World Health Organisation as a key strategy to ensuring equitable access to hearing care [16]. The other advantage of this approach is improved quality and cultural competence of hearing service delivery [16]. Evidence supports the benefits of task shifting, with similar outcomes related to disability-adjusted life years averted [17] while being cost-effective and scalable [18].

In hearing health care, there is an opportunity to explore task shifting to audiology students. Beyond closing the hearing healthcare gap, this opportunity presents as an enhanced learning experience for the students to allow for reflection on the link between course content and practice [19], as well as an interprofessional education experience as students work together in the community to provide hearing healthcare to clients [20]. These types of experiential learning are associated with benefits in improved academic outcomes, subject matter understanding, critical thinking and problem solving, collaborative practice patterns as well as interpersonal and personal development [19,20]. They are also a particularly useful opportunity for students to interact with older adult clients, a population group who have an increasing prevalence of hearing loss as they age [21]. This kind of intergenerational connection between younger and older adults has been explored in the research and found to promote intrapersonal development between the two generations and more positive attitudes towards older adults by the younger generation [19,22]. In Australia, there are audiology courses in each state and the development and implementation of intergenerational programs that can enhance student learning experiences and access to hearing healthcare are warranted.

Beyond the involvement of audiology students, mobile health (mHealth) digital technologies can support effective task shifting in hearing health care through easy-to-use equipment that does not require extensive training to operate. Existing mHealth technologies have enabled trained non-specialists to provide hearing screening [23] and diagnostic testing on the go comparable to that of hearing specialists in a clinic [24], using automated testing procedures with quality controls like noise-level monitoring. An example of such technology is the Sound Scouts tablet-based app to screen hearing loss. The initial app gamified speech-in-quiet, speech-in-noise, and tones-in-noise tests and achieved sufficiently high specificity and sensitivity to provide hearing screening in children [25]. The Sound Scouts team have recently adapted this app to include an automatic audiometer which was found to produce valid and reliable hearing thresholds with appropriate calibration of the Sennheiser HD 300 headphones [26]. Training of nonspecialists to administer the app outside of audiology clinics is a strategy worthy of exploration to make hearing screening more accessible and support earlier intervention in this population. Sound Scouts has strong potential as a tool to train audiology students to screen for hearing loss in older people as it has already enabled over 230,000 hearing checks by non-specialist parents and teachers in children.

Further to this, examination of other ear issues like eardrum abnormalities or excess earwax provides more a in-depth understanding of potential hearing loss. However, acquiring training and equipping users with traditional otoscopes can present a practical and financial barrier to the successful task-shifting of hearing care. To overcome this financial barrier, a low-cost, solar-powered pocket otoscope (Arclight) has been developed and tested in both low to middle- and high-income countries [27,28]. In a comparative mixed-methods analysis, Arclight was equally effective in objective diagnostic performance and time to make a diagnosis compared to a traditional otoscope. Where cost presents a significant barrier to training and equipping for hearing healthcare, Arclight offers an affordable alternative that maintains full functionality.

Within communities around the world, Meals on Wheels is a trusted service known for low-cost meal deliveries to older adult clients and a strong reliance on volunteers to support their operations. In Australia, the Meals on Wheels New South Wales (NSW) branch has approximately 14,000 volunteers serving 4,500,000 meals annually to over 22,000 clients. With the organisation’s trademarked slogan “More Than Just a Meal”, Meals on Wheels provides a range of services which includes wellbeing checks performed by trained volunteers at every meal delivery [29]. Currently, the Meals on Wheels wellbeing check does not include a hearing health check (hearing screening and otoscopy). Embedding routine hearing checks using Sound Scouts and Arclight, and education on hearing loss within existing Meals on Wheels services, offers a holistic and low-cost approach to providing support for vulnerable older adults at increased risk of hearing loss.

In the proposed study, we will partner with Meals on Wheels NSW, Sound Scouts, the Arclight project team, as well as Master of Clinical Audiology students, to deliver a co-designed, novel model of hearing care outside of a clinical setting. The aim of this study is to pilot this model of care, known as the SOUND-BITES program, across Meals on Wheels sites in metropolitan Sydney to evaluate its acceptability, feasibility and preliminary efficacy.

## Materials and methods

### Study design

This pilot study aims to implement and evaluate the SOUND-BITES program using a mixed-methods approach. Ethics approval has been obtained by the Macquarie University Human Research Ethics Committee (ID: 16818) on 11 December 2025. A co-design process involving focus groups, workshops and interviews with staff, volunteers and clients from Meals on Wheels was conducted to inform the design of this pilot study. A separate manuscript is in preparation to present the details of the co-design process.

### Research participants

The research participants will be clients from Meals on Wheels NSW and their household members. Clients will be recruited across eight Meals on Wheels NSW sites involved in the pilot study in metropolitan Sydney. These include Parramatta Food Services, The Village Chef, Cumberland Food Services, Holdsworth Street Community Centre, Liverpool Meals on Wheels, Bankstown Food Services, Myrtle Cottage, and Nepean Food Services. Based on discussion with Meals on Wheels NSW, household members such as a spouse, would be included to support the reach and impact of SOUND-BITES. It was also determined that a feasible target sample size for this study would be 60 volunteers and 600 clients and household members across these locations, with every volunteer recruited, supporting the hearing checks of 10 research participants (clients and/or household members).

To recruit volunteers, flyers will be distributed through participating Meals on Wheels sites with information about the study and participation details. Volunteers will register their interest by contacting the research team through the contact details provided in the flyer. The research team will assess eligibility, with eligible volunteers meeting the following criteria i) is a registered volunteer at one of the participating Meals on Wheels locations, ii) is aged 18 year or older, and iii) is willing and available to attend training sessions and deliver the hearing health checks and education. Volunteers will be excluded if they lack sufficient English fluency to provide their own informed consent and understand their involvement in the study. Eligible volunteers will receive a Participant Information and Consent Form to review and ask any questions before providing written consent to participate. The research team will organise online or in-person events for live questions and answers as needed with interested volunteers for each Meals on Wheels site. Another Participant Information Form will be circulated to participating volunteers about an optional post-program interview. This interview aims to evaluate the volunteer experience of the SOUND-BITES program and determine the program’s acceptability and feasibility.

To recruit clients and their household members, service coordinators at each Meals on Wheels site will initially pre-screen their client database for eligible participants. Any participants that are recorded as having a known diagnosis of cognitive impairment such as dementia will not be contacted about the study. For the remaining clients, they will initially receive a flyer to raise awareness about the study followed by an information pack that will be delivered along with their next meal delivery service. The information pack will include a brochure with summarised information about the study, an Expression of Interest Form and a Participant Information and Consent Form. The client and/or their household member can express an interest in participating in the study. Meals on Wheels volunteers will collect Expression of Interest Forms on their next visit. Clients and/or household members who express interest will be contacted by their respective Meals on Wheels service coordinator to discuss their participation, confirm their eligibility and answer any questions. Eligible clients will be a registered client at one of the participating Meals on Wheels locations, while eligible household members are eligible if they live in the same household as a Meals on Wheels client. Additional eligibility criteria for both clients and household members include being aged 50 years or older, having sufficient English fluency to provide own informed consent and an understanding of their involvement in the study. The age of eligibility for this study has been selected to align with recommendations that hearing loss screening should be implemented from age 50 [30]. In addition to a known diagnosis of cognitive impairment, clients and household members will also be excluded if they are currently using a hearing aid and/or cochlear implant for >2hrs per day on average. This criterion supports the inclusion of research participants who are ‘low’ or inconsistent hearing device users. This categorisation is based on usage scales applied in standardised measures like the International Outcomes Inventory for Hearing Aids (IOI-HA) and Abbreviated Profile of Hearing Aid Benefit (APHAB) questionnaire where ≤1hr of device use is ’low’ [31–33] and knowledge that hearing aid use is often over-estimated by at least 1hr [34]. Eligible clients and/or household members who wish to participate in the study will then be asked to complete the Participant Information and Consent Form. In this consent form, clients and/or household members can also indicate their consent to be contacted about an optional interview after their hearing health check. Upon receipt of the consent form, a hearing health check will be scheduled with the client and/or household member.

### Master of clinical audiology students

The opportunity to take part in this research study will be presented as a clinical placement option for Master of Clinical Audiology students at Macquarie University. Based on preferences and availabilities of students, they will be allocated to the study by the University Clinical Placement Supervisor.

A summary of the roles and contributions of the Meals on Wheels clients and household members, volunteers and Master of Clinical Audiology students is shown in Table 1.

**Table 1:** Summary of Meals on Wheels client and household member, volunteer, and audiology student involvement in the SOUND-BITES program.

	Meals on Wheels Client and Household Member	Meals on Wheels Volunteer	Audiology Student
**Recruitment**	1. Receive information flyer 2. Receive booklet with more detailed information	1. Information flyers 2. Q&A events as required online or in-person	Students will be invited to the project as part of their Clinical Placement and allocated to the project by the Course Convener
**Consent**	Written consent form completed prior to home visit	Written consent form completed prior to training session commencement	Written consent form completed prior to training session commencement
**Training**	N/A	3-hour training session. Covers: • Education about hearing health • Overview of the hearing assessment • Overview of providing feedback on clients’ hearing assessment results • Overview of the education component • SOUND-BITES study administration	1.5-hour training session Covers: • Meals on Wheels Services • Conducting a hearing health assessment • Providing feedback about a hearing assessment • Providing education about hearing health • SOUND-BITES study administration
**Home Visits – SOUND-BITES Hearing Health Check**	Receive 1 home visit lasting approximately 30 minutes Receives a Hearing Health Check including: • Otoscopic examination • Hearing assessment using the Sound Scouts app • Feedback on results of assessments • Education around importance of addressing hearing health	Attend ~10 home visits with Meals on Wheels clients over 2–4 half day shifts (~ 4 hours/day) or 2 full day shifts (~6.5 hours/day) or a combination of half and full day shifts (~16 hours total) Main roles: • Provide key relationship link with Meals on Wheels Client • Support students and clients with hearing health check assessments & education	Attend visits with Meals on Wheels clients over 2–4 half day shifts (~ 4 hours/day) or 2–6 full day shifts (~6.5 hours/day) or a combination of half and full day shifts (~40 hours total, including travel time) Main roles: • Conduct otoscopic examination of the ear • Conduct Sound Scouts Hearing Assessment • Provide results and education
**Baseline Questionnaires**	Paper-based questionnaire including: • Demographics and medical history • Hearing Handicap Inventory for the Elderly – Short Form • Speech, Spatial and Qualities of Hearing Scale 12 • Falls History • EQ-5D-5L	Paper-based questionnaire • Demographics • Technology Experience	N/A
**Qualitative Interviews**	Optional telephone or online semi-structured interview lasting ~30 min. To occur within 4 weeks of their home visit. Only a sub-sample will complete the semi-structed interview. The interview will assess the acceptability and feasibility of the SOUND-BITES Program. Approximately 10–14 Meals on Wheels clients will complete the interview.	Optional telephone or online semi-structured interview lasting ~30 min. To occur within 4 weeks of their participation in the home visits. The interview will assess the acceptability and feasibility of the SOUND-BITES Program. Approximately 10–14 Meals on Wheels volunteers will complete the interview.	N/A
**Follow-Up Questionnaire (6 months following Home Visit)**	Paper-based questionnaire including: • Demographics and medical history • Hearing Handicap Inventory for the Elderly – Short Form • Speech, Spatial and Qualities of Hearing Scale 12 • Falls History • EQ-5D-5L • Behaviours after hearing health check	N/A	N/A

### Volunteer and student training program

In-person training will be provided to both volunteers and students involved in the study. For volunteers, they will participate in a 3-hour training course that will cover:

- General education about hearing health
- Overview of the hearing health check using the Sound Scouts app and Arclight device
- Overview of the hearing check feedback given to clients and/or household members
- Overview of the client and/or household member hearing health education
- Administrative procedures that volunteers need to follow to deliver the SOUND-BITES program. This includes information about participant identification numbers, confidentiality and recording of data/information.

The training serves to raise awareness about hearing health among the volunteers and allows themselves to familiarise with the SOUND-BITES processes. During this pilot study, volunteers are not expected to perform any of the hearing health checks. At the conclusion of the training, the volunteers will complete a brief, anonymous questionnaire to capture demographic information and one question about their experience using a computer, tablet or smartphone to capture familiarity with technology use.

For the Master of Clinical Audiology students, they will participate in a 1.5-hour training course. As the students will be performing the hearing health checks in this pilot study, their training is more practical and builds on their existing clinical training. Therefore, it will cover:

- Overview of Meals on Wheels services and the role they play in the community and the value of community-delivered models of hearing care
- Overview and practice conducting a hearing health check using the Sound Scouts app and Arclight device
- Practice providing hearing check feedback to Meals on Wheels clients and/or household members based on the Sound Scouts app and Arclight outputs
- Practice providing client and/or household member education about hearing health
- Administrative procedures that the students need to follow to deliver the SOUND-BITES program. This includes information about study ID’s, confidentiality, data collection and safety procedures.

### SOUND-BITES program delivery

Following the recruitment and training of Meals on Wheels volunteers and Master of Clinical Audiology students, a hearing health check for consenting clients and/or household members will be scheduled by the respective service coordinator at each participating Meals on Wheels site. During the scheduled visit, a pair of audiology students and one volunteer will attend the client’s home to complete the hearing health check. This check will start with an otoscopic examination of the ear canal and tympanic membrane using Arclight’s integrated magnifying loupe and ear speculum attachment site. Observations about the ear canal and ear drum, presence of earwax, ear infection, exostoses and foreign objects will be documented in a standardised form (S1 Appendix). For clients and/or household members whose otoscope examination reveals any abnormalities, they will be advised to see their General Practitioner for further management. Following the otoscopy is an 8-to-10-minute hearing screening test using the Sound Scouts app, which will be preloaded to a study iPad. Students will set up a new ‘player’ account for each client and prepare the test to ensure standardisation across clients in terms of headphones used and tone selection. A headphone sound check will be conducted and instructions on how to complete the assessment will be provided to the client and/or household member. Equipment will be cleaned immediately before and after use for each client and/or household member. For the hearing screening test, clients and/or household members will wear over the ear headphones (Sennheiser HD 400S) and respond to tones heard by pressing a button on the iPad screen at four frequencies – 500, 1000, 2000 and 4000 Hz to 20dB HL. All clients and/or household members will complete a trial so they can practise responding to the tones prior to completing the real test. Following the test, the Sound Scouts app will produce an audiometric output for the left and right ear. For each ear, there will be a recommendation of “Refer” or “Pass” and a hearing number reported in decibels (dB) to indicate level of difficulty hearing in noisy places. The hearing number can range from −10–19 (little to no trouble), 20–34 (some trouble), 35–49 (a lot of trouble), 50–64 (severe trouble), 65–79 (extreme difficulty), and 80 or higher (may not be able to understand) [35]. In addition, an audiogram will be produced to illustrate hearing thresholds at each of the four frequencies. Audiology students will discuss the results of the hearing screening test with the client and/or household member and provide the relevant education. If hearing loss is indicated, the client and/or household member will be recommended to seek a diagnostic assessment for hearing loss and provided with a list of local audiology services, who provide services for the Australian Hearing Services Program – a government-funded program that provides access to subsidised hearing services and devices to eligible Australians with hearing loss [36]. Volunteers can participate as much as they are comfortable with during this visit.

At the end of the visit, clients and/or household members will be given a survey to complete within two weeks in their own time. The survey will take approximately 15–20 minutes to complete and will include questionnaires on demographics, medical history, hearing loss, hearing handicap, falls history and quality of life. Participants will be provided with a reply-paid envelope to return the survey to Meals on Wheels volunteers during subsequent meal delivery runs. The service coordinators at each Meals on Wheels site will then post the envelopes and surveys to the Macquarie University Research Team for data entry into REDCap. Overall, it is proposed that Meals on Wheels volunteers will be involved with baseline hearing health checks across two to four half day shifts of approximately four hours each, two full day shifts of approximately 6.5 hours each or a combination of half and full day shifts, up to a maximum of 16 hours. The audiology students will be involved in four to ten half day shifts, or two to six full day shifts up to a maximum of 40 hours as required for their clinical placement.

### SOUND-BITES program outcomes measures

A range of outcome data will be collected through the SOUND-BITES program. This includes clinical data from the Arclight otoscopy examination and Sound Scouts hearing screening test results; quantitative data from the baseline and 6-months post-program surveys and qualitative data from the 4-weeks post-program client and/or household member and volunteer interviews. Each of these outcome measures are described in more detail below.

### Clinical data

Clinical data will be captured in a REDCap form. Using Arclight, the otoscopy data will capture observations about the ear canal and ear drum, presence of earwax, ear infection, exostoses and foreign objects. Any other relevant observations can be noted (S1 Appendix). The audiometric data obtained using the Sound Scouts hearing screening app will capture hearing thresholds for each ear at 500, 1000, 2000 and 4000 hertz, down to 20 decibels hearing level. Upon completion of the hearing screening test, the suggested hearing status (normal hearing or potential hearing loss) will be documented. Participants suggested to have a hearing loss will be provided referral details as described earlier.

### Quantitative data

A survey will be administered at baseline and 6-months post-program. It will capture demographics, medical history, falls history and responses to the Hearing Handicap Inventory for the Elderly – Short Form (HHIE-S) [37] (primary outcome), The Line tool (hearing intervention readiness) [38], Speech, Spatial and Qualities of Hearing Scale 12 (SSQ-12) [39], and EuroQol (EQ-5D-5L) [40] (Table 2). In addition, the 6-month post-program questionnaire will evaluate post-program help-seeking behaviours. That is, for participants who sought help for their hearing, they can complete questions related to who they consulted with about their hearing loss, their reasons for seeking help, and specify any outcome/s of seeking help such as receiving a hearing aid. For participants who did not seek help for hearing loss identified during the SOUND-BITES program, they will be asked about their reasons for not seeking help and about any intentions for future help-seeking.

**Table 2:** Outcome measures in SOUND-BITES.

Category	Items/Instruments
**Demographics**	Age, sex, marital status, living situation, ethnicity, education
**Medical History**	Health conditions including cardiovascular disease, diabetes, arthritis, kidney disease, cancer, vision loss, hearing loss. If hearing loss is present, there will be questions about the use of hearing devices.
**Falls History**	Two questions on the presence of any falls in the last 12 months and the frequency of these falls
**Hearing-related Scales**	The Line tool – asking 1) ‘How important is it for you to improve your hearing right now?’, and 2) ‘How much do you believe in your ability to use a hearing aid?’. Participants are asked to select a number on an unmarked visual analogue scale between 0 (not important/unable) to 10 (of highest importance/able) to reflect their own situations. Hearing Handicap Inventory for the Elderly – Short Form (HHIE-S). This is a 10-item questionnaire for screening hearing disability or handicap in older adults across five social or situational items and five emotional response items. A total HHIE-S score of ≥8 indicates the presence of a hearing handicap. Speech, Spatial and Qualities of Hearing Scale 12 (SSQ-12). This questionnaire evaluated hearing disability across three domains – speech hearing, spatial hearing and qualities of hearing. Response options are presented as an 11-point Likert scale where a higher score indicates greater ability (less disability).
**Quality of Life and Cost-Benefit**	EuroQol EQ-5D-5L and Visual Analogue Scale. This evaluates five dimensions – mobility, self-care, usual activities, pain/discomfort and anxiety/depression. Respondents tick the box next to the statement that best corresponds to their current health state. For the Visual Analogue Scale, respondents indicate their health at the time of completion as a number between 0 (worst health) to 100 (best health).

### Qualitative data

Participating Meals on Wheels clients and/or household members and volunteers will be given the opportunity to participate in an optional audio-recorded one-on-one interview. Participants will be given the option of a telephone interview or online interview using Zoom based on their preference and accessibility needs. For the clients and/or household members, this interview would be scheduled after their hearing health check and involve a mix of multiple-choice questions to evaluate the quality of the overall program, the hearing health check and the education component as well as open-ended questions to reflect on the most useful component of the program and feedback for improvement (S2 Appendix). For the volunteers, their interview would be scheduled at the end of the program when no additional client hearing health checks are needed. These interviews will ask questions around the perceived value of the SOUND-BITES program, comfort and capability to deliver the program with and without the Master of Audiology students, and feedback to improve the program (S3 Appendix). Qualitative insights will inform the acceptability and feasibility of SOUND-BITES (primary endpoints).

### Analyses

Descriptive statistics including summarising binary and categorical outcomes (e.g., help-seeking behaviours) using frequency and percentages will be used to describe data collected at baseline and 6-months post-program. Means and standard deviations or median and interquartile ranges will be used to summarise continuous outcomes, e.g., quality of life scores. Chi-squared test or Fisher’s exact test will be applied to compare binary outcomes and independent two sample t-tests for continuous outcomes. The primary endpoints of this pilot study include the acceptability rate of the program and help-seeking among participants (feasibility). These endpoints will be estimated with 95% confidence intervals. Generalised linear models will be fit to investigate differences at baseline and 6-months post-program in, e.g., change in attitudes to loss of hearing and hearing handicap (HHIE-S) scores. Adjustments will be made for potential confounders including age and sex. Economic evaluation using incremental cost of hearing screening will be measured using the EQ-5D-5L scale, which is also a cost-utility analysis instrument.

Audio-recorded interview data from participating clients and/or household members and volunteers will be transcribed using intelligent verbatim in Microsoft Word. Each transcript will be manually checked by a research team member against the audio recording for accuracy. Cleaned transcripts will be returned to the respective participant who will be given two weeks to review the transcript and provide any corrections or additional information. Finalised transcripts will be imported into the latest version of NVivo and through deductive content analysis, coded into the Theoretical Domains Framework (TDF) [41]. The TDF is an ideal framework as it is commonly used to evaluate the implementation of interventions. Prior to coding, the research team will develop a coding guideline to increase the reliability of coding between the two researchers who will be involved in this process (i.e., coders). These coders will then familiarise themselves with the participants’ responses in the transcripts, consider the relevance of the responses to definitions of the TDF domains and then attribute the responses to the most relevant domain/s. The text coded to the TDF domains should reflect an overarching theme. Throughout the coding process, the two coders will meet to discuss their codes to reach an agreement. A third researcher will be engaged if consensus between the two coders is not achieved. Data analysis and data collection via interviews will occur concurrently to determine the point of data saturation. This is the point where the research team does not identify new insights about the SOUND-BITES program from the interviews [42]. According to a systematic review evaluating sample sizes for saturation in qualitative research, saturation is reached after 9–17 interviews [42].

### Status and timeline

This study has commenced recruitment of Master of Clinical Audiology students on 01/03/2026. No Meals on Wheels volunteers or clients and/or household members have been recruited into the study yet and no data has been collected. Participant recruitment is expected to be completed by 12/10/2026. Full data collection is anticipated to be completed by 30/04/2027 with subsequent phases dedicated to data cleaning, analysis and generation of meaningful results. Preliminary results are expected by 01/09/2027 and these may be shared at relevant scientific conferences. Full study outcomes will be submitted for peer-reviewed publication upon completion.

## Discussion

Hearing loss is a global priority with a financial burden of US$1 trillion per year [43] strategies to increase access to hearing healthcare could significantly alleviate some of this burden. In Australia, task shifting to Meals on Wheels NSW volunteers and Master of Audiology students presents an untapped opportunity to improve access to and management of hearing health for vulnerable older adults. Moreover, the partnership with Sound Scouts and the Arclight team leverages existing evidence-based and user-friendly mHealth technologies to facilitate the upskilling of these trained non-specialists. The valuable knowledge that will be gained from this pilot study will aid in identifying best practices, developing standard operating procedures, and determining the most effective way to deliver and evaluate the SOUND-BITES program on a wider scale.

SOUND-BITES is a promising strategy to not only increase timely identification and education about hearing loss in older adults but will also enable them to live in their own homes with dignity, independence, and in optimal health. However, this pilot study is not without limitations. It has been designed to assess feasibility and acceptability and therefore may not be powered to detect significant changes in quantitative outcomes. As hearing checks are conducted in Meals on Wheels client homes, environmental factors such as background noise, will expectedly vary from home to home and may affect the results of the checks. There may also be challenges with navigating audiology student availability as student involvement is restricted to a 32-week clinical placement period. Moreover, training will need to be provided for every new placement student joining the project and consideration to develop pre-recorded training content may be needed for ongoing sustainability. Similar considerations may also be required if there is a high turnover of Meals on Wheels volunteers or regular intake of new volunteers. Nonetheless, this pilot study will serve to identify the limitations of SOUND-BITES to guide further refinement and will provide the foundation for conversations with the broader Meals on Wheels network and other audiology programs to roll-out the program across other states and territories in Australia. Findings from this pilot study will be disseminated through peer-review journals and conferences as well as community networks.

## Supporting information

S1 Appendix SOUND-BITES hearing assessment data collection. (DOCX)

S2 Appendix Client interview guide. (DOCX)

S3 Appendix Volunteer interview guide. (DOCX)
