# Supplementary material for: Embedding routine hearing health checks within existing Meals on Wheels services – A protocol for the SOUND-BITES Program pilot study
Source: PLoS One. 2026 Jul 14;21(7):e0354082. doi: 10.1371/journal.pone.0354082 (PMC13367903; doi:10.1371/journal.pone.0354082)
Supplement: S1 Appendix — (DOCX) [file pone.0354082.s001.docx]

S1 Appendix. SOUND-BITES Hearing Assessment Data Collection

| Participant ID: |  |
| --- | --- |
| Participant Year of Birth: |  |
| Date of Assessment: |  |
| Time of Assessment: |  |
| Name of Students and Volunteer at Assessment |  |

| **Task** | **Completed by (Name)** | **Results/Comments** |
| --- | --- | --- |
| 1. Consent form completed and signed |  |  |
| 2. Otoscope Examination |  | Normal (ear canal and ear drums clear)  Eardrum abnormality  Non-occluding ear wax  Impacted ear wax  Ear Infection  Exostoses  Foreign object  Other: |
| 3. Sound Scouts Audiometer Assessment |  | Normal Hearing  Hearing Loss |
| 4. Results explained |  |  |
| 5. Education provided |  |  |
| 6. Booklet provided |  |  |
| 7. Questionnaires provided |  |  |
