# Supplementary material for: Embedding routine hearing health checks within existing Meals on Wheels services – A protocol for the SOUND-BITES Program pilot study
Source: PLoS One. 2026 Jul 14;21(7):e0354082. doi: 10.1371/journal.pone.0354082 (PMC13367903; doi:10.1371/journal.pone.0354082)
Supplement: S2 Appendix — (DOCX) [file pone.0354082.s002.docx]

S2 Appendix. Client Interview Guide

Thank you for taking the time to participate in the SOUND-BITES research project. The purpose of today’s interview is to understand your experience receiving the hearing health check with Meals on Wheels as part of the SOUND-BITES project.

First, we have some demographic questions to ask you.

1. What is your year of birth?
2. What is your gender?
3. What is your postcode?
4. What is your marital status?
5. What country were you born in?

Interview Questions:

1. Overall, how would you rate the quality of the SOUND-BITES program?

- Very high quality
- High quality
- Moderate quality
- Low quality
- Very low quality

1. How would you rate the quality of the hearing health check?

- Very high quality
- High quality
- Moderate quality
- Low quality
- Very low quality

1. How would you rate the quality of the hearing education provided?

- Very high quality
- High quality
- Moderate quality
- Low quality
- Very low quality

1. Do you feel that it is appropriate to include this program within the existing Meals on Wheels services that are currently provided to you?

- Yes
- No

1. Why/Why not?
2. Was it worth your time doing the program?

- Yes
- No

1. Would you recommend the SOUND-BITES program to family/friends?
2. What was the most useful components of the SOUND-BITES program?
3. Do you have any suggestions to improve the SOUND-BITES program?
