# Supplementary material for: Embedding routine hearing health checks within existing Meals on Wheels services – A protocol for the SOUND-BITES Program pilot study
Source: PLoS One. 2026 Jul 14;21(7):e0354082. doi: 10.1371/journal.pone.0354082 (PMC13367903; doi:10.1371/journal.pone.0354082)
Supplement: S3 Appendix — (DOCX) [file pone.0354082.s003.docx]

S3 Appendix. Volunteer Interview Guide

Thank you for taking the time to participate in the SOUND-BITES research project. The purpose of today’s interview is to understand your experience receiving the hearing health check with Meals on Wheels as part of the SOUND-BITES project.

First, we have a few demographic questions.

1. What is your year of birth?
2. What is your gender?
3. What country were you born in?
4. What is your marital status?
5. How long have you been a volunteer with MoW?

Interview Questions:

1. Overall, do you think the SOUND-BITES program is a valuable addition to the services offered by Meals on Wheels? Why/Why not?
2. Would you feel comfortable delivering the hearing assessment on the iPad without the support of the Audiology students? Why/Why not?
3. Would you feel comfortable delivering the hearing education without the support of the Audiology students? Why/Why not?
4. Would you recommend Meals on Wheels clients to take part in the SOUND-BITES program? Why/Why not?
5. How do you think other volunteers at your service who haven’t taken part in the SOUND-BITES program would feel about taking part in the future? Do you have any suggestions on how we could attract more volunteers to take part in the program?
6. What do you think are the most beneficial aspects of the SOUND-BITES program?
7. Do you have any other suggestions to improve the program?
